# Valproic-induced Hyperammonemic Encephalopathy in a Known Case of Epilepsy

**DOI:** 10.7759/cureus.1557

**Published:** 2017-08-10

**Authors:** Syed F Imam, Omair ul haq Lodhi, Rizwan Zafar, Saneeya Nasim, Waseem T Malik

**Affiliations:** 1 Department of Internal Medicine, Shifa International Hospital, Islamabad, Pakistan; 2 Shifa College of Medicine, Shifa International Hospital, Islamabad, Pakistan; 3 Department of Neurology, Shifa International Hospital, Islamabad, Pakistan

**Keywords:** valproic acid, hyperammonemia, encephalopathy, ammonia

## Abstract

Valproic acid, a broad-spectrum anticonvulsant drug, commonly causes elevated ammonia levels, which is usually asymptomatic in most cases. On rare occasions, potentially fatal hyperammonemia-induced encephalopathy can occur. We present a case of a 24-year-old female who presented to the emergency department with status epilepticus that was being managed with valproic acid. Further workup was done because of prolonged postictal state, which revealed increased ammonia levels; she was eventually diagnosed with valproic-induced hyperammonemic encephalopathy. Discontinuing valproic acid resulted in drastically improved symptoms and a gradual decline in ammonia levels. A clinician should be aware of rare drug adverse effects and drug interactions to conclusively reach the correct diagnosis. A prolonged postictal state should warrant further workup to rule out other possible etiologies.

## Introduction

Valproic acid (VPA), a branched short-chain fatty acid, raises the gamma-aminobutyric acid (GABA) level by inhibiting GABA transaminase. It is used for the treatment of various seizures, neuropathic pain, migraine headaches, and psychiatric disorders, such as bipolar disorders, schizoaffective disorders, and social phobias. VPA requires carnitine to enter the liver mitochondria and go into the *β*-oxidation process. Therefore, in cases administrating a high therapeutic dose chronically or an acute overdose of VPA, carnitine depletion can occur, which results in VPA accumulating outside the mitochondria. Consequently, this would increase the *ω*-oxidation pathway and increase 4-en-VPA concentration. This process results in abnormal ammonium elimination in the urea cycle. Additionally, VPA toxic metabolites can inhibit carbamoyl phosphate synthetase that catalyzes the first step of the urea cycle, which is carbamoyl phosphate formation from ammonia, and thus, also increasing ammonia levels [1].

As ammonia levels increase in the brain, the extracellular level of glutamate increases as the glutamate uptake is inhibited. Glutamate triggers N-methyl-D-aspartate receptors (NMDA receptors), decreasing the threshold of seizures and thereby increasing the risk. Concurrently, glutamine synthesis increases and builds up in the astrocytes, which result in swelling of the astrocytes and cerebral edema. Furthermore, ammonia also has a detrimental effect on the neurons by blocking the Kreb's cycle [2].

Although VPA-induced hyperammonemia is a very common adverse effect, it rarely results in encephalopathy [3]. We present a case of a 24-year-old female who was diagnosed with hyperammonemia-induced encephalopathy secondary to the acute valproic acid administration for the treatment of status epilepticus. The hyperammonemia resolved after discontinuation of the valproic acid.

## Case presentation

A 23-year-old female, a known case of epilepsy since the age of 11 years with good drug compliance on carbamazepine and clonazepam, presented to the emergency department (ED) with status epilepticus. It initially began as a continuous jerky movement of the right half of her body, including her face, arm, and leg. Since the previous night, the jerky movements occurred after variable intervals and the duration would range from a few minutes to an hour. There was no associated aura, loss of consciousness, tongue biting, rolling of eyes, urinary or bowel incontinence, blank stares, vomiting, or fever before she presented to the ED.

On initial examination, the patient presented with tonic clonic movements of all her limbs and was unconscious. Her pulse was 99 beats per minute with SpO2 at 96%, blood pressure (BP) was 110/80 mmHg, temperature was 98.5° F, and respiratory rate was 22 breaths per minute.

After the introduction of an intravenous line, she was given levetiracetam followed by phenytoin with no benefit. She was then given intravenous valproic acid. Her seizures stopped eventually; however, she ended up in a confused state. This was initially thought to be a postictal stage. After no improvement in the next 24 hours, further workup was done, including EEG, MRI, liver function test, lumbar puncture with cerebrospinal fluid analysis and culture, and an autoimmune workup. All tests came back normal except for the EEG, which was abnormal, and ammonia levels, which were drastically elevated. The laboratory investigations are shown in Tables 1-2. The patient was eventually diagnosed with valproic-induced hyperammonemic encephalopathy (VHE).

**Table 1:** Serial Laboratory Investigations

Laboratory Investigations	Day 1	Day 2	Day 3	Day 4	Day 5	Day 6	Normal Values
Serum sodium	139	138	140	136	136	138	(136 - 144 mEq/L)
Serum potassium	4.2	4.1	4.4	3.6	3.8	3.7	(3.7 - 5.2 mEq/L)
Serum chloride	103	102	116	105	105	109	(101 - 111 mEq/L)
Serum bicarbonate	24	22	18	23	21	21	(22 - 28 mEq/L)
Serum creatinine	0.49	0.63	0.74	0.61	0.60	0.58	(0.8 -1.2 mg/dL)
Serum urea	13.8	16.3	21.4	10.7	4.28	12.84	(7 - 20 mg/dL)
Alanine aminotransferase	-	24	21	23	27	22	(7 - 56 U/L)
Aspartate aminotransferase	-	19	18	24	33	27	(10 - 40 U/L)
Alkaline phosphatase	-	120.5	115.1	98.3	74.5	73.3	(44 - 147 U/L)
Gamma-glutamyl trasferase	-	73	69	68	63	60	(9 - 48U/L)
Total bilirubin	-	0.36	0.28	0.27	0.24	0.30	(0.3 - 1.9 mg/dL)
Direct bilirubin	-	0.29	0.20	0.17	0.09	0.15	(0 - 0.3 mg/dL)
Hemoglobin	14.10	12.60	10.70	13.87	12.3	11.4	(12 -15.5 g/dL)
White blood cell, Total	8,600	8,800	9,700	6,900	5,500	5,500	(4,500 - 11,000 /μL)
Serum ammonia	-	-	378	301	-	-	(15 - 45 μg/dL)
C-reactive protein	-	-	-	15.56	-	-	(0 - 3.0 mg/L)
Valproic acid, Total	-	-	-	75.88	-	-	(50 - 125 μg/mL)
Phenytoin	-	-	-	9.15	-	-	(10 - 20 μg/mL)

**Table 2:** Serological, CSF, and Immunological Investigations CSF: cerebrospinal fluid; PCR: polymerase chain reaction

Laboratory Investigation	CSF Culture	Hepatitis B Surface Antigen	Hepatitis C Surface Antibodies	CSF-PCR Herpes Simplex 1 & 2	CSF-PCR Mycobacterium Tuberculosis	Leucin-rich glioma inactivated protein 1	Contactin-associated protein 2
Results:	No growth detected after 4 days of intubation	Not reactive	Not reactive	Not detected	Not detected	Negative	Negative

After the diagnosis was established, the valproic acid was discontinued on day 4. In the next 24 hours, the patients’ confusion improved significantly. Additionally, L-carnitine was also administered to the patient. Ammonia levels gradually started decreasing. When the patient was stable, she was discharged on her previous home medications with a follow-up plan of care.

## Discussion

VPA-induced encephalopathy can occur due to multiple reasons that include elevated ammonia levels or a toxic metabolite of VPA, 2–en-vpn, which induces cerebral edema. The latter can have a prolonged period of recovery depending on the severity of edema due to the long half-life of 2-en-vpn. In our patient, recovery from the stupor state was immediate after VPA discontinuation (< 24hrs) and CT was negative for cerebral edema, favoring the VHE diagnosis [4].

The incidence of hyperammonemia with VPA can be as high as 100%. As it does not always result in encephalopathy and is mostly asymptomatic, withholding of the drug is not always needed. VPA can cause increased ammonia levels with or without elevated liver enzymes; therefore, plasma ammonium concentration should be measured in clinically suspected cases of VHE, even if liver function tests are normal. Our patient had increased ammonia levels with normal liver function tests [3].

Ammonia levels in VPA toxicity rise in a dose-dependent manner and other risk factors can have an additive effect on this rise, including malnutrition, female gender, concomitant use of other anti-epileptic drug’s (AEDs), and antipsychotics [2, 5]. Yoshiaki, et al. concluded that enzyme-inducing AEDs have a more potent effect in increasing ammonia levels than non-inducer AED’s [5]. Our female patient was on long-term carbamazepine and also received phenytoin in the emergency department, which could explain her immediate rise in ammonia and resultant confusion.

VPA-induced hyperammonemia is usually asymptomatic, but in rare cases, it can present with fatigue, stupor, focal neurological signs and symptoms, and a decreased seizure threshold. Very rarely, it can cause asterixis, aggression, vomiting, ataxia, and coma. Our patient only had stupor and no other signs or symptoms [6].

Postictal confusion, which can last from minutes to rarely days, can mask the clinical signs of encephalopathy and delay the diagnosis. Therefore, a patient in a postictal state with a prolonged period of confusion should warrant further workup for alternate diagnoses, such as metabolic abnormalities, drugs, or infections. In our case, there was compelling evidence for VPA-induced encephalopathy as her ammonia levels were markedly elevated and, after discontinuation of the offending agent, her level of consciousness improved drastically. Moreover, a thorough workup consisting of serological, radiological, and immunological tests were done to rule out any other possible etiologies [7].

The definitive treatment of VHE involves withdrawing the offending agent, e.g., valproic acid. Other measures that are proven to be beneficial include sodium benzoate, sodium phenylacetate, and L-carnitine. In severe refractory cases, dialysis has been proven to be effective in decreasing ammonia levels. Discontinuing VPA resulted in a prompt recovery in less than 24 hours in our patient [2].

## Conclusions

We conclude from our experience that if there is a strong clinical suspicion of an alternate diagnosis, the clinician should be open-minded and further investigate the possibility of a concurrent illness. Additionally, the physician should be aware of rare drug adverse effects and drug interactions to conclusively reach the correct diagnosis as early as possible to avoid any unnecessary tests. This would also aid in formulating an accurate management plan to provide the patient with the best plan of care. As in our case of VHE, a mere discontinuation of the offending drug, valproic acid, had a drastic effect on recovery.
